# X-Ray and Radium Protection Committee

**Published:** 1921-05-28

**Authors:** 


### X-ray and Radium Protection Committee.

The preliminary meeting of the above Committee took place at 1 Wimpole Street, W. (by the kind permission of the Royal Society of Medicine), on the 18th inst. The Committee is composed of radiologists, physicists, pathologists, and others, nominated by the Electrotherapeutic Section of the Royal Society of Medicine, the British Association for the Advancement of Radiology and Physiotherapy, the Institute of Therapy, the Institute of Physics, the Roentgen Society, and the National Physical Laboratory, and is under the chairmanship of Sir Humphry Rolleston, K.C.B., M.D., F.R.C.P.

The intention of the Committee is to issue very shortly a considered statement as to the urgency of affording adequate protection to the worker, indicating the best means whereby this may be carried out. For this purpose a Sub-Committee has been appointed. The further intention of the Committee is to collect data bearing on the effect of irradiation, with particular reference to protection; to carry out special research, if necessary; to act in a consultative and, possibly, advisory capacity, and to publish from time to time such reports as are considered necessary.

